# Instructing item-specific switch probability: expectations modulate stimulus–action priming

**DOI:** 10.1007/s00426-021-01641-z

**Published:** 2022-01-18

**Authors:** Janine Jargow, Uta Wolfensteller, Christina U. Pfeuffer, Hannes Ruge

**Affiliations:** 1 Faculty of Psychology, Technische Universität Dresden, 01062 Dresden, Germany; 2 Department of Psychology, Albert-Ludwigs-Universität Freiburg, 79098 Freiburg, Germany

## Abstract

Both active response execution and passive listening to verbal codes (a form of instruction) in single prime trials lead to item-specific repetition priming effects when stimuli re-occur in single probe trials. This holds for task-specific classification (stimulus–classification, SC priming, e.g., apple–small) and action (stimulus–action, SA priming, e.g., apple–right key press). To address the influence of expectation on item-specific SC and SA associations, we tested if item-specific SC and SA priming effects were modulated by the *instructed* probability of re-encountering individual SC or SA mappings (25% vs. 75% *instructed* switch probability). Importantly, the experienced item-specific switch probability was always 50%. In Experiment 1 (*N* = 78), item-specific SA/SC switch  expectations affected SA, but not SC priming effects exclusively following active response execution. Experiment 2 (*N* = 40) was designed to emphasize SA priming by only including item-specific SC repetitions. This yielded stronger SA priming for 25% vs. 75% expected switch probability, both following response execution as in Experiment 1 and also following verbally coded SA associations. Together, these results suggest that SA priming effects, that is, the encoding and retrieval of SA associations, is modulated by item-specific switch expectation. Importantly, this expectation effect cannot be explained by item-specific associative learning mechanisms, as stimuli were primed and probed only once and participants experienced item-specific repetitions/switches equally often across stimuli independent of instructed switch probabilities. This corroborates and extends previous results by showing that SA priming effects are modulated by  expectation not only based on experienced item-specific switch probabilities, but also on mere instruction.

**Supplementary Information:**

The online version contains supplementary material available at 10.1007/s00426-021-01641-z.

## Introduction

### Item-specific SA and SC priming

Responding to a stimulus according to the current task demands can entail two processes (Horner & Henson, 2011, 2012; Moutsopoulou et al., 2015). On the one hand, a stimulus (e.g., car) can require a task-specific semantic classification (e.g., small/large). On the other hand, a stimulus can require an action (e.g., left/right manual response). This then potentially leads to the formation of stimulus–classification (SC) associations (e.g., car–large) and stimulus–action (SA) associations (e.g., car–right; Moutsopoulou et al., 2015). After such item-specific SC and SA associations have been formed, they can be tested via item-specific repetition priming effects by comparing item-specific switches and repetitions of SC/SA mappings between a stimulus’ prime trial and probe trial (see, e.g., Henson et al., 2014; Logan, 1988, 1990, for earlier accounts of stimulus–response associations and repetition priming). The retrieval of SC/SA associations leads to faster responses and fewer errors when the item-specific required classification/action repeats compared to when the required classification/action does not match the one implied by the previously established SC/SA association for that stimulus (item-specific SC/SA priming effect; Horner & Henson, 2011, 2012; Moutsopoulou et al., 2015; Pfeuffer et al., 2017). More specifically, there is evidence for facilitation after SC/SA repetition (e.g., Horner & Henson, 2011, 2012) as well as for interference when SC/SA mappings switch from prime trial to probe trial (Horner & Henson, 2011, 2012; Moutsopoulou et al., 2015).

In the item-specific priming paradigm used in the present study, participants were to classify everyday objects by pressing one of two response keys. Everyday objects are displayed only once as a prime and once as a probe with a lag of two to seven trials in between (Moutsopoulou et al., 2015; Pfeuffer et al., 2017; Pfeuffer, Hosp et al., 2018). Item-specific SC and SA mappings are orthogonally repeated/switched between an item’s prime and probe trial, allowing for independent assessment of item-specific SC and SA priming effects in probe trial reaction times and error rates (comparing item-specific switches and repetitions of SC/SA mappings between prime and probe).

SA and SC associations were previously reported to be independent components of stimulus–response (SR) associations (Horner & Henson, 2009; Moutsopoulou & Waszak, 2013; Moutsopoulou et al., 2015; Pfeuffer et al., 2017). That means that reaction time (RT) and error rates independently increased for item-specific SC and SA switches (between prime and probe) compared to repetitions (Horner & Henson, 2009; Moutsopoulou & Waszak, 2013; Moutsopoulou et al., 2015; Pfeuffer et al., 2017).

Importantly, by assessing item-specific priming effects, Pfeuffer et al. (2017), Pfeuffer, Hosp et al. (2018) and Pfeuffer, Moutsopoulou et al. (2018) found that both, SC and SA associations were formed not only by active response execution (called ‘executed blocks’), but also by passive listening to verbal instructions (called ‘verbally coded blocks’) denoting the relevant classification and the respective action for a given stimulus during a prime trial. As an example, participants viewed the image of an apple and heard the verbal codes ‘small, right’ via headphones. This corresponds with findings from other experimental paradigms consistently showing that item-specific SR associations can be formed following single-trial response execution and following mere single-trial instruction without response execution (Cohen-Kdoshay & Meiran, 2007; Liefooghe & De Houwer, 2018; Liefooghe et al., 2012; Meiran & Cohen-Kdoshay, 2012; Meiran et al., 2017; Oshrit & Meiran, 2009; Ruge & Wolfensteller, 2010; Ruge et al., 2018, 2019; Wenke et al., 2007).

### Expected switch probability based on experience

Previous studies found that expectations based on previous experience modulated SR associations and conflict (Aben et al., 2017; Abrahamse et al., 2016; Leboe et al., 2008). Moreover, context-specific modulation of performance based on previous experiences (though not item-specific priming effects) has been reported (Crump & Logan, 2010; Leboe et al., 2008; Thomson et al., 2013, 2014). For instance, Crump and Logan (2010) showed that priming effects differed depending on location cues. When location was predictive of high or low task switch probability, task switch costs were reduced/increased based on experienced task switch probabilities, respectively. Thus, there is evidence that experienced probabilities influence performance.

Furthermore, experienced probabilities of conflict or task switches can become associated with specific items (Chiu & Egner, 2017; Chiu et al., 2020; Jacoby et al., 2003; Leboe et al., 2008). In these conflict or task switching studies, specific stimuli were presented multiple times with specific proportions of task switches or conflict (incongruent mappings). For stimuli with experienced high probabilities of task switches/conflict, the effects of task switches/conflict decreased compared to stimuli with low probability of task switches/conflict (e.g., item-specific proportion congruency effect: e.g., Bugg et al., 2008; Jacoby et al., 2003; Schmidt & Besner, 2008); or item-specific switch probability effect (e.g., Chiu & Egner, 2017; Chiu et al., 2020; Kang & Chiu, 2021). These effects of experienced probabilities can be explained by associative learning mechanisms. That is, the experienced probability (over multiple trials) of a stimulus being paired with a task switch/conflict leads to adaption processes like contingency learning (Blais & Bunge, 2010; Jacoby et al., 2003; Schmidt, 2013; Schmidt & Besner, 2008).

Finally, there is evidence that experienced switch probability influences item-specific SC/SA priming effects (Pfeuffer et al., 2020) even when stimuli are primed and probed only once. Across the prime and probe instances of stimuli, Pfeuffer et al. (2020) realized different experienced item-specific SC/SA repetition/switch probabilities in different groups of participants (e.g., in Exp. 2: frequent condition: item-specific SC repetition and SA switch > 70%; infrequent conditions: 3 other combinations of item-specific SC and SA repetitions/switches < 30% in total). Most importantly, an item-specific repetition/switch was only experienced once per stimulus in the probe when the effect of this item-specific repetition/switch was tested. Therefore, item-specific switch probabilities were only realized across the entire list of stimuli. That means, item-specific associative learning of classifications/actions or repetition/switch probabilities was impossible. Nevertheless, when SC/SA switches were frequent (across different previously encountered stimuli), the respective item-specific SC/SA priming effects for novel primed and probed stimuli were reduced as compared to when SC/SA repetitions were frequent. That is, there were modulatory effects of switch probability experienced across different previously encountered stimuli on the size of priming effects in probe trials.

As participants were unable to explicitly report repetition/switch proportions afterward, Pfeuffer et al. (2020) argued that their effects were based on implicit learning processes (see also Blais et al., 2012; Crump & Logan, 2010; Thomson et al., 2014). Thus, SC/SA priming effects were modulated based on participants’  expectation based on experiences across different previously encountered stimuli. This modulation of item-specific priming effects by experienced switch probability was similarly observed after both, active response execution and passive listening to verbal codes during the corresponding prime trials (Pfeuffer et al., 2020).

### The present study: expected switch probability based on instruction

Importantly, so far, the influence of expectations on item-specific priming effects (i.e., on the encoding and/or retrieval of corresponding associations) has only been tested based on expectations induced by own previous experience. In contrast, the influence of explicit expectations on SC/SA priming in the absence of  expectation-inducing experience (e.g., based on mere instruction) has not been assessed. Specifically, it is unknown whether and how an explicit instruction that item-specific repetitions/switches are frequent, which induces an expectation, affects item-specific SC/SA priming effects. Here, we thus investigated whether item-specific SC/SA priming effects are also modulated by expected switch probabilities induced by mere instruction. Moreover, in contrast to previous studies, we assessed expected switch probabilities that changed frequently and unpredictably across the course of the experiment. Doing so, we aimed to assess whether participants were able to rapidly adapt (i.e., by changing their SC/SA associations and showing reduced/increased SC/SA priming effects) to frequently varying item-specific switch expectations based on instructions (i.e., explicit knowledge).

In other research fields, expected probabilities based on prior experience versus instruction were investigated, for instance, in the context of the description–experience gap in risky decision making (Dutt et al., 2014; Hau et al., 2008; Hertwig & Erev, 2009; Park et al., 2021). In fact, participants seem to treat experienced and instructed probabilities differently leading to contrasting decisions (Hau et al., 2008). One key difference between description (instruction) and experience is that people tend to give less weight to small probabilities (rare events) when they decide based on experience compared to description (Hertwig & Erev, 2009). Based on these findings, it seems possible that expectations based on instruction (present study) versus based on experience (Pfeuffer et al., 2020) differently modulate item-specific priming effects.

Additionally, as priming can be described as the facilitation of different processes (e.g., perception, classification, action), it is most interesting that also verbal codes (i.e., instruction) can lead to item-specific priming effects as previously mentioned (Pfeuffer et al., 2017; Pfeuffer, Moutsopoulou et al., 2018). While item-specific priming effects based on execution were extensively investigated (e.g., Horner & Henson, 2011, 2012; Moutsopoulou et al., 2015), it is not quite clear yet how verbal codes lead to the formation of SR associations. Recent studies on priming effects of motor imagery (Liefooghe et al., 2021; Palenciano, 2021) suggest that motor imagery might be a contributing factor, but, for instance, subvocal rehearsal and visual imagery also cannot be excluded as of now.

On the one hand, previous studies found strong similarities between SC and SA associations formed by active task execution and by verbal coding (Pfeuffer et al., 2017; Pfeuffer, Hosp et al., 2018). But on the other hand, priming effects were found to be larger after execution compared to after verbal coding (Pfeuffer et al., 2017, 2020; Pfeuffer, Hosp et al., 2018) and, specifically, only the multiple execution but not multiple prime trials of verbal coding of SC/SA mappings led to increased priming effects. (Pfeuffer, Moutsopoulou et al., 2018). This points towards essential differences between the mechanisms underlying these types of priming. Most importantly for the present study, priming effects based on execution and verbal coding were similarly influenced by expectations based on experienced switch probability (Pfeuffer et al., 2020). Nevertheless, to rule out that only one type of priming might be susceptible to instructed switch proportions, we additionally compared execution and verbal coding. That is, we were interested in whether processes of SC and SA priming based on execution and verbal coding are equally susceptible to modulation by expectations based on instruction. Alternatively, verbally coded SR associations might be more susceptible to expectations based on instructed switch probability than already executed SR associations simply, for instance, due to a common declarative representational format.

The present study set out to address these questions based on a modified version of the item-specific priming paradigm previously used by Pfeuffer and colleagues.

First, instead of establishing different switch probabilities through actual experience, in the present study item-specific instructed switch probabilities (25% vs. 75%) were induced via cues. Experienced item-specific switch probability remained constant at 50% throughout the experiment. That is, unpredictably, for half of the stimuli the item-specific SC/SA mappings repeated between the stimulis’ prime and probe, whereas they switched for the other half of stimuli. Second, item-specific instructed switch probabilities changed randomly across blocks allowing for within-subject comparisons. These two procedural modifications enabled us to explicitly test the hypothesis that item-specific SC/SA priming effects are modulated by item-specific switch xpectations induced by instruction (i.e., by explicit knowledge) and are flexibly adapted from block to block.

To our knowledge, no study has previously assessed the influence of item-specific switch expectations derived from explicit knowledge (mere instruction) on item-specific priming and SC/SA encoding/retrieval. Nevertheless, our hypothesis that explicit knowledge and resulting expectations can modulate item-specific priming effects is somewhat supported by previous studies regarding modulations of response conflict. These studies suggest that trial-wise instructions might be sufficient to modulate response conflict and to induce expectation-based proportion congruency effects in the Simon task (Desender, 2018; Wühr & Kunde, 2008) and in the Stroop task (Bugg & Smallwood, 2016; Bugg et al., 2015; Entel et al., 2014). Importantly, however, in these studies (with the exception of a single experiment in Bugg et al., 2015), induction blocks were used, where the instructed switch probability was experienced, to ensure that instructions seemed valid to participants (Bugg & Smallwood, 2016; Bugg et al., 2015; Entel et al., 2014). However, by confounding instructed and experienced switch probability and testing stimuli multiple times, these studies could not differentiate between influences of experience and mere instruction (i.e., explicit knowledge). As mentioned above, this confound was avoided in the present study, as instructed item-specific switch probability (25% or 75%) changed randomly across prime-probe blocks, while the experienced item-specific switch probability was held constant at 50%. Therefore, we were able to investigate the modulation of item-specific SA and SC priming effects (i.e., corresponding SC/SA associations) based on expected switch probabilities induced by mere instruction which were not strengthened by experienced switch probabilities.

To summarize, the present study addressed two research questions concerning the modulation of SC and SA priming effects by expectation:

If SC and SA associations were encoded and retrieved solely based on mnemonic processes, SC and SA priming effects should remain unaffected by merely expected (instructed), but never experienced item-specific SC/SA switch probabilities. Vice versa, a modulation of SC/SA priming effects by merely instructed item-specific SC/SA switch probabilities would imply that participants adapted to expectations induced by instruction/explicit knowledge. We hypothesize that item-specific priming effects can be modulated on the basis of not only previous experience (Pfeuffer et al., 2020), but also expectations derived from mere instruction and will be stronger under instructed 25% switch probability, compared to instructed 75% switch probability.

We additionally assessed if SC/SA associations formed by different types of prime trials (executed vs. verbally coded) were similarly modulated by instructed switch probability (interactions of prime type, instructed switch probability, and action/classification). On the one hand, one could expect that both prime types are similarly affected by expectation, as Pfeuffer et al. (2020) found no difference between these prime types regarding the influence of  expectation based on experienced switch probability. On the other hand, differences between the prime types (Pfeuffer et al., 2017, 2020; Pfeuffer, Hosp et al., 2018) could influence the susceptibility to instructed switch probability. Thus, observing similar or different effects of instructed switch probability depending on prime types would also be informative regarding the mechanisms of priming (facilitation of perception, classification or action) which can be modulated by expectations derived from instruction/explicit knowledge.

## Experiment 1

In Experiment 1, we investigated if instructed switch probability (25% vs.75%) affected SC/SA priming effects in two groups. In both groups, participants were instructed that classification (SC group) or action (SA group) could switch from an item’s prime trial to its probe trial (lag 2–7 trials) with a probability of either 25% or 75%. Instructions were delivered via cues preceding the item (‘instructed switch probability’) during prime trials—with experienced switch probability during probe trials being held constant at 50%. We hypothesized that the instructed switch probability would affect item-specific SC/SA priming effects according to the instructed switch probability group: SC but not SA priming effects should be specifically weaker after an instructed 75% (vs. 25%) SC switch probability (SC group). Conversely, SA but not SC priming effects should be specifically weaker after an instructed 75% (vs. 25%) SA switch probability (SA group). That is, we hypothesize interactions of group × instructed switch probability × classification and group × instructed switch probability × action.

This SC/SA specificity hypothesis is based on the results of Pfeuffer et al. (2020). They found that SC/SA switch probability experienced for other stimuli did indeed specifically influence either SC/SA priming effects. However, in the present study, participants would have to use the instructed switch probability information specifically either for SC or for SA associations during an already complex paradigm. Considering the overall high cognitive load, it is questionable if participants would be able to do that. We therefore also tested the alternative non-specificity hypothesis that instructed switch probability would affect SC/SA associations irrespective of group (i.e., instructed switch probability × classification and instructed switch probability × action). To foreshadow the results, we indeed found that participants did not use the instructed switch probability specifically for SC or SA associations.

### Methods

#### Participants

Sample size was determined based on the effect sizes of previous similar studies on item-specific priming (Pfeuffer et al., 2017, 2020). Most importantly, a previous study investigated the interaction of experienced switch probability and SC/SA priming (Pfeuffer et al., 2020).

The means of the effect sizes of Pfeuffer et al. (2020) for experience-based expectation modulation of SC priming (*f* = 0.360) and SA priming (*f* = 0.176) were calculated. Using these priors for power calculations using G*Power (Faul et al., 2007), a sample size of at least 38 subjects per group is sufficient to gain a power above 80% at an alpha level of 5% for the detection of within-subject interaction effects in repeated measures ANOVAS.

A total of 83 participants (56 female, 27 male) took part in the experiment. We adopted the exclusion criteria of Pfeuffer et al. (2020). One subject was excluded based on not having performed the task properly as evidenced by his/her post-experiment comments. Two participants were excluded due to insufficient performance (error rates > 3 SDs above mean). Two further participants were excluded, because after exclusion of trials with response omissions and errors in primes and corresponding probes, the data did not contain enough trials per cell for the analyses (at least 12 probe trials per condition). After these exclusions, the final sample comprised 78 participants (52 female, 26 male; mean age = 24.0, SD = 4.0, range 19–39; 5 left handed). Participants provided written informed consent and received 8 Euro per hour or course credit. Participants were randomly assigned to either the SC group (*N* = 38 after exclusions, 26 female, 12 male, 2 left-handed, mean age: 23.9, SD = 4.3) or to the SA group (*N* = 40 after exclusions, 26 female, 14 male, 3 left-handed, mean age: 24.1, SD = 3.7).

#### Stimuli and apparatus

Participants were tested individually in assessment rooms where they viewed stimuli presented on a standard LCD screen (17 inch) with a viewing distance of approximately 60 cm. Manual responses were executed with their left and right index fingers resting on two keys of a standard PC keyboard (D and K) placed in front of them with a key distance of approximately 10 cm. Response keys were marked with coloured patches. Participants wore headphones throughout the experiment.

**Fig. 1:**
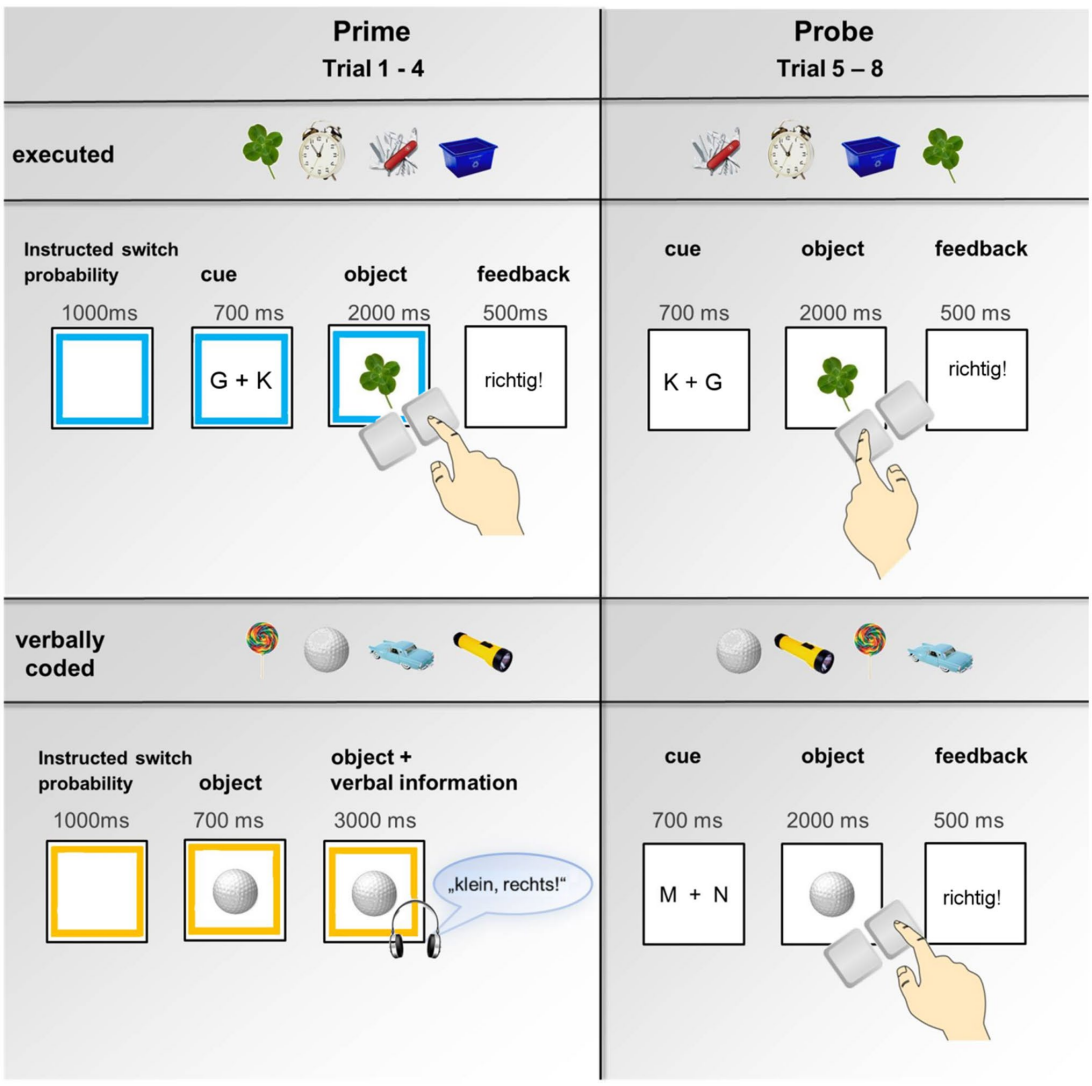
Experimental paradigm: trial structure of executed and verbally coded blocks. Four prime trials (executed vs. verbally coded) are followed by the corresponding four probe trials (executed). In executed trials, participants actively classified the object and responded according to the task cue with left or right key presses in both primes and probes. Task cues for the size classification task were ‘K + G’ or ‘G + K’ corresponding to the first letters of the German words for ‘small’ (K—‘klein’) and ‘large’ (G—’groß’). The task cue for the mechanism task was either ‘M + N’ or ‘N + M’ corresponding to the first letters of the German words for ‘mechanical’ (‘mechanisch’) and ‘non-mechanical’ (‘nicht-mechanisch’). In verbally coded prime trials, participants passively listened to a verbal instruction while viewing the object on the screen. They then classified the same stimuli actively in the corresponding probe trials. Each stimulus appeared only once as a prime and once as a probe (lag 2–7 trials). Paradigm and figure adapted from Pfeuffer et al. (2017)

We adopted a modified version of the SC/SA priming paradigm described previously in Pfeuffer et al. (2017) and Moutsopoulou et al. (2015). The experiment comprised executed (active responding during prime and probe) and verbally coded blocks (passive listening during the prime and active responding during the probe) that were intermixed. Each block consisted of a prime phase (trial 1–4), a probe phase (trial 5–8), and a memory recall phase (trial 9; see Fig. 1 for the trial structure). In each block, four new images were presented in prime trials and every image was only repeated in the corresponding four probe trials of the same block. That is, each image appeared only once as a prime and once as a probe throughout the experiment. One of the four images was presented in the recall trial of the same block.

As stimuli, we used 512 images of everyday objects (256 pixels × 256 pixels, about 8° visual angles). The stimulus set was the same as in Pfeuffer et al. (2017) adapted from sets by Brady et al. (2008) and Moutsopoulou et al. (2015). An additional 24 images were used for an initial practice phase preceding the main experiment. Stimuli were presented in random order.

Participants performed one of two classification tasks (in executed prime trials and all probe trials). In the size task, they indicated whether or not a depicted object would fit into a reference box (dimensions 37.5 cm × 30 cm × 14.5 cm). This box was shown to participants and positioned in the assessment room. In the mechanism task, participants indicated whether or not the depicted object was mechanic or not. The task cue for the size tasks was either ‘K + G’ or ‘G + K’ corresponding to the first letters of the German words for ‘small’ (K—’klein’) and ‘large’ (G—’groß’). The task cue or the mechanism task was either ‘M + N’ or ‘N + M’ corresponding to the first letters of the German words for ‘mechanical’ (‘mechanisch’) and ‘non-mechanical’ (‘nicht-mechanisch’). Objects were classified by pressing the left or right key corresponding to the spatial position of the intended classification as indicated by the task cue.

#### Design and procedure

Participants received general instructions regarding the experimental procedure. They were instructed to always put their index fingers on the response keys prior to the start of a block to avoid delays in probe trials following verbally coded prime trials. Also, they were specifically told not to perform any responses during verbally coded prime trials, but to only attend to the spoken verbal codes (i.e., instructions). For executed primes and probes, they were instructed to respond as quickly and correctly as possible. Participants were also instructed to attend to and memorize item-specific classifications and actions during prime trials for the recall trial at the end of the block.

Most importantly, according to their group membership, participants were informed that either item-specific classification (SC group) or action (SA group) would switch with a certain probability (25% vs. 75%) according to the colour of the frame surrounding stimuli during prime trials (SC/SA instructed switch probability). Participants were instructed to use this information to optimize their performance.

**Fig. 2:**
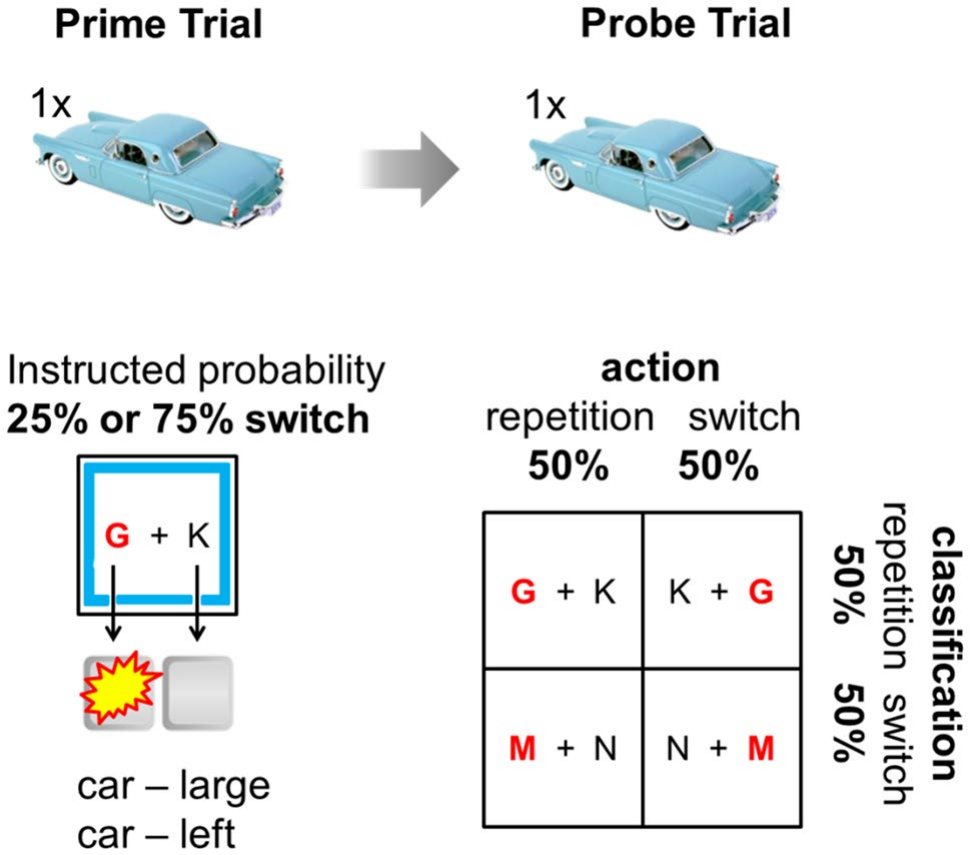
Schematic overview of instructed switch probability and actual item-specific action and classification repetitions and switches between the prime and probe of a specific stimulus. In prime trials, differently coloured frames served as instructed switch probability cues denoting the probability with which item-specific SC or SA mappings would (supposedly) switch from prime trials to probe trials (note that actual switch probability was always 50%). For both prime types (executed and verbally coded), each combination of action and classification repetition/switch occurred equally often. Task cues for the size classification task were ‘K + G’ or ‘G + K’ corresponding to the first letters of the German words for ‘small’ (‘klein’) and ‘large’ (‘groß’). The task cue for the mechanism task was either ‘M + N’ or ‘N + M’ corresponding to the first letters of the German words for ‘mechanical’ (‘mechanisch’) and ‘non-mechanical’ (‘nicht-mechanisch’). The red bold letter denotes the correct answers regarding classification (letter) and action (spatial position). Paradigm and figure adapted from Pfeuffer et al. (2017) (colour figure online)

In a practice run, participants got a step-by-step instruction to familiarize them with the block types and they performed 4 complete practice blocks (2 blocks with executed primes and 2 blocks with verbally coded primes). The main experiment consists of 128 prime-probe blocks (64 blocks with executed prime trials and 64 blocks with verbally coded prime trials (intermixed, random order). Participants were informed about the block type (executed vs. verbally coded) prior to the start of an upcoming block: A slide with the text ‘next block: visual’ (German: ‘Nächster Block: Visuell’) or ‘next block: verbal’ (German: ‘Nächster Block: Verbal’) was presented. Therefore, participants did know if they had to respond from the beginning of the block (executed) or first attend do the verbal instruction (verbally coded) and only respond to later (probe) trials. On the same slide, participants were reminded which coloured frame represented which instructed switch probability. Each block consisted of four prime trials, followed by four corresponding probe trials (lag 2–7 trials). Item-specific SC and SA mappings were randomly repeated or switched (50:50) between an item's prime and probe trial resulting in four item-specific switch conditions (classification repetition–action repetition, classification repetition—action switch, classification switch–action repetition, classification switch–action switch; see Fig. 2). Orthogonal switches of action and classification allowed an independent investigation of SA and SC priming effects.

##### Prime trials (trial 1–4)

Prime trial structure was tailored to our needs by presenting cues during the prime trials, which denoted the instructed SC/SA switch probability for the current prime-probe block. SC/SA switch probability was indicated via the colour of frames (blue vs. orange) indicating a high/low probability of SC/SA switches depending on participants group (see Pfeuffer et al., 2019, for an example using the same context cues to signal the intentional settings lying vs. truth telling).

As we outlined in the introduction, it is well known that participants can associate location, but also other cues (here: different frame colours) with different switch probabilities. To prevent our 75% and 25% switch instruction cues from being associated with the experienced 50% switch probability during probes, we presented the cue during the prime trials. Additionally, we did not have a strong a priori expectation if the encoding (in prime trials) and/or the retrieval (in probe trials) of SC/SA associations would be modulated by instructed switch probability. Therefore, in order not to miss potential encoding effects, we presented the instructed switch probability cues already during the prime phase.

Each prime trial started with a coloured frame (duration 1000 ms) which denoted the item-specific switch probability (SC vs. SA depending on participants’ group; SC/SA instructed switch probability), that is, the (only alleged) probability for a switch in the item specifically required classification or action for the stimulus in its later probe trial. The coloured frame (orange vs. blue) for instructed SC/SA probability stayed on screen during the subsequent presentation of the task cue (700 ms) and the stimulus (until response or max. 2000 ms) in the executed prime trials and during the presentation of stimulus and verbal code (3700 ms in total) in verbally coded prime trials. In executed blocks, during prime trials, participants actively classified stimuli by pressing the key corresponding to the correct classification as fast as possible. Feedback (‘correct!’/‘richtig!’ for correct responses, ‘error!’/‘Fehler’ in red ink, for incorrect responses, ‘too slow!’/‘zu langsam!’ in red ink, for response omissions) was presented for 500 ms directly after response execution. In the prime trials of verbally coded blocks, the stimulus was presented for an initial 700 ms. After that, the stimulus was presented for a further 3000 ms and verbal codes regarding classification and action were presented via voice recording playbacks (1.8–2.3 s) with an emotionally neutral female voice (e.g., ‘small, left’/‘klein, links’).

##### Probe trials (trial 5–8)

Probe trials were all executed (both for stimuli primed by execution and verbal coding), beginning with a 700 ms task cue, followed by stimulus presentation until response execution (max. 2000 ms), and 500 ms feedback directly after response execution.

##### Recall trial (trial 9)

In the memory recall trial, participants were asked to report the classification and action mapped to a randomly selected stimulus verbally coded or executed in one prime trial of the same block. To ensure participants’ attention to all prime instances, we asked them to try to memorize prime trial SA and SC mappings (i.e., to attend to the classification and action they heard or executed, the first time, a picture was presented and to try to memorize them). The memory recall trial was not speeded, and participants were instructed to focus on high accuracy. For memory recall, the screen turned grey, and participants were presented with one of the four objects of that block. First, for classification memory recall, the question ‘category?’ (German: ‘Kategorie?’) above the image prompted participants to press the key on the keyboard corresponding to the classification of the displayed object during the prime trial (‘K’ for ‘klein’, small/‘G’ for ‘groß’, large, ‘M’ for ‘mechanisch’, mechanic and ‘N’ for ‘nicht-mechanisch’, non-mechanic). Second, the prompt ‘reaction R/L?’ (German: ‘Reaktion R/L?’) indicated that participants should perform the action (left vs. right key press) that the object had been associated with during its prime. Memory recall trials were only introduced to ensure participants’ attention to the prime SC and SA mappings. Memory recall results are presented in the Appendix.

#### Data analyses

Analyses were conducted using SPSS Statistics version 27 (IBM SPSS Statistics, Armonk, NY). Performance data from both classification tasks (size and mechanism) and both manual responses (left and right) were pooled. In all statistical analyses reported, an alpha level of 0.05 was used.

Prime trial analysis can be found in the supplementary material.

For probe analysis, probe trial percentages of errors  (PEs) and mean RTs were submitted to a 2 × 2 × 2 × 2 × 2 mixed-design ANOVA with the between-subject factor group (SC vs. SA) and the within-subject factors prime type (executed vs. verbally coded), instructed switch probability (25% vs. 75%), classification priming (repetition vs. switch), and action priming (repetition vs. switch). Significant interactions were followed up via one-tailed paired *t* tests (according to stated hypotheses) if not noted otherwise.

### Results

#### Probe trial analyses

For the probe trial analyses, we excluded all probes with omitted or erroneous prime responses (4.6%) and verbally coded primes with overt responses (0.2%). Additionally, probe response omissions were excluded (0.7%). For RT analysis, erroneous probe trials (7.8%) and outliers (RTs deviating more than 3 SDs from the corresponding individual cell mean; 0.9% of the remaining probe trials) were excluded. For RTs, these criteria led to the exclusion of 14.6% of probe trials. All ANOVA results can be found in Table S1 in the Supplementary Material.

##### Probe RT analysis

First, we wanted to be sure that the basic pattern of item-specific classification and action priming effects based on execution and verbal coding were replicated, before we addressed the additional effect of instructed switch probability.

*Replication of previous effects* Indeed, we replicated the relevant main effects reported in Pfeuffer et al. (2017): We found a main effect of classification priming reflecting that participants were slower when the item-specific classification switched rather than repeated between prime and probe (*M*_Priming_ = 42 ms), *F*(1,76) = 152.41, *p* < 0.001, *η*_*p*_^2^ = 0.67. The main effect of action priming was also significant (*M*_Priming_ = 8 ms), *F*(1,76) = 8.80, *p* = 0.004, *η*_*p*_^2^ = 0.10, with longer RTs for item-specific action switches as compared to repetitions. We also found an effect of prime type, *F*(1,76) = 42.58, *p* < 0.001, *η*_*p*_^2^ = 0.36, constituting that probe responses were significantly faster after verbally coded primes compared to executed primes (*M*_diff_ = 37 ms).

In correspondence with results reported in one previous study on item-specific priming (Pfeuffer et al., 2020, which manipulated experienced switch probability), classification priming and action priming interacted significantly, *F*(1,76) = 4.40, *p* = 0.039, *η*_*p*_^2^ = 0.06 (but see Horner & Henson, 2009, 2011; Moutsopoulou & Waszak, 2013; Moutsopoulou et al., 2015; Pfeuffer et al., 2017 for contrary findings not suggesting an interaction). Exploring this interaction, we conducted two-tailed paired *t* tests. Interestingly, action priming effects were only significant after classification repetitions (*M*_Priming_ = 14.6 ms), *t*(77) = 3.47, *p* < 0.001, *d* = 0.38, but not after classification switches (*M*_Priming_ = 4.2 ms), *t*(77) = 1.18, *p* > 0.120, *d* = 0.13.

In accordance with previous studies (Pfeuffer et al., 2017; Pfeuffer, Hosp et al., 2018), we found an interaction of prime type and classification priming (executed: *M*_Priming_ = 63 ms; verbally coded: *M*_Priming_ = 21 ms), *F*(1,76) = 50.07, *p* < 0.001, *η*_*p*_^2^ = 0.40, and an interaction of prime type and action priming (executed: *M*_Priming_ = 16 ms; verbally coded: *M*_Priming_ = − 0.1 ms), *F*(1,76) = 8.00, *p* = 0.006, *η*_*p*_^2^ = 0.09. Classification priming was significant for both executed and verbally coded primes, *t*s(77) ≥ 5.3, *p* ≤ 0.001, *d* ≥ 0.60, whereas action priming effects were only significant after executed, *t*(77) = 3.86, *p* < 0.001, *d* = 0.44, but not after verbally coded primes, *t*(77) < 0.03, *p* = 0.490, *d* = 0.00.

*Modulation by instructed switch probability* Surprisingly, there was an additional main effect of instructed switch probability, *F*(1,76) = 5.09, *p* = 0.027, *η*_*p*_2 = 0.06, with higher error rates after instructed 25% switch probability (*M* = 8%) compared to instructed 75% switch probability (*M* = 7.4%).

**Fig. 3:**
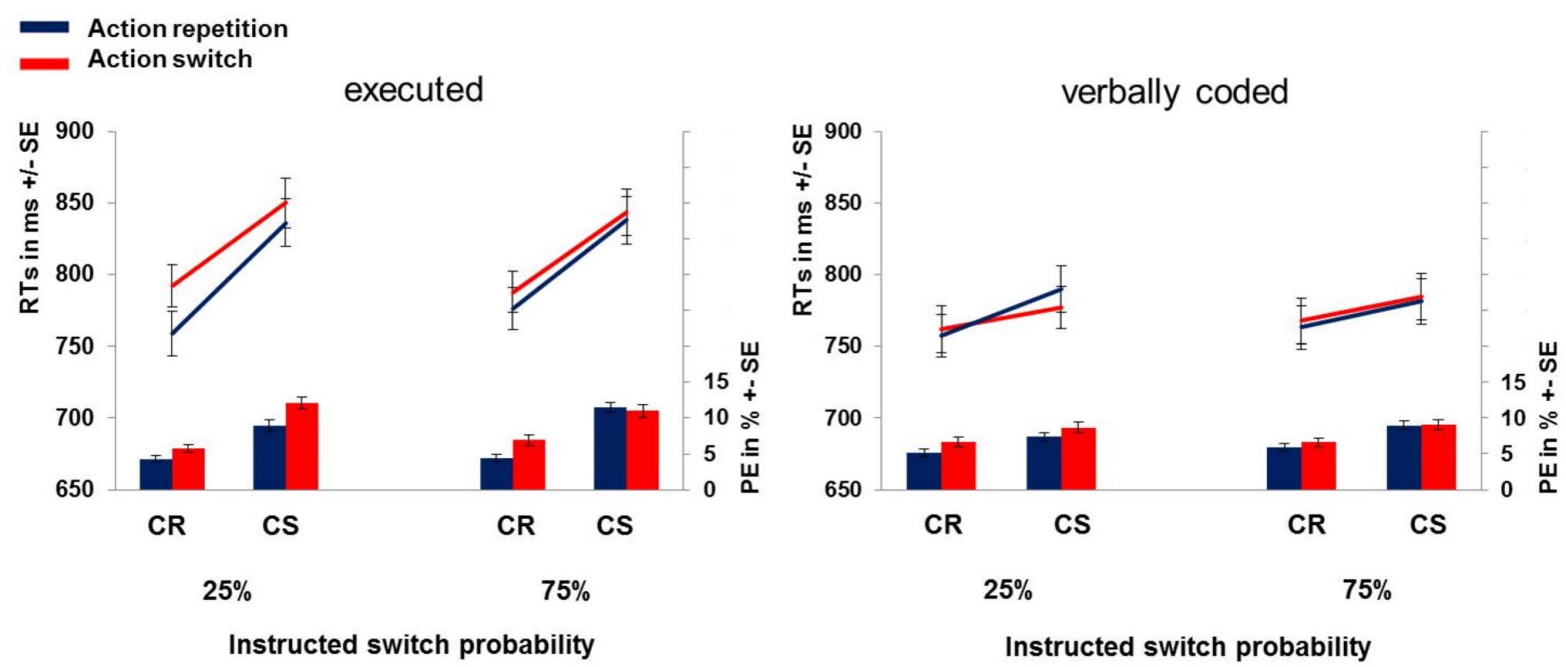
Probe results of Experiment 1: mean RTs (lines) and PEs (bars) shown separately for executed and verbally coded blocks, the instructed switch probability (75% or 25%), and the actual classification/action switch or repetition condition. *CR* classification repetition, *CS* classification switch

In accordance with our key hypothesis, the interaction of prime type and action priming was further qualified by a three-way interaction with instructed switch probability*, F*(1,76) = 6.40, *p* = 0.014, *η*_*p*_^2^ = 0.08 (see Fig. 3). There were significant execution-based action priming effects after instructed 25% switch probability, (*M*_Priming_ = 23.7 ms) *t*(77) = 4.51, *p* < 0.001, *d* = 0.51, and after instructed 75% switch probability, (*M*_Priming_ = 9.7 ms); *t*(77) = 1.83, *p* = 0.036, *d* = 0.21, which were significantly different in size depending on the instructed switch probability, *t*(77) = 2.16, *p* = 0.017, *d* = 0.24. In contrast, verbal code-based action priming was not significantly modulated by instructed switch probability, (*M*_diff_ = 7.8 ms), *t*(77) = 1.15, *p* = 0.127, *d* = 0.13. There was no interaction of action priming and instructed switch probability that was further modulated by group, *F*s(1,76) ≤ 0.86, *p*s ≥ 0.358, *η*_*p*_^2^ ≤ 0.01.

Also contrary to our hypotheses, there was no significant interaction of instructed switch probability with classification priming, *F*(1,76) = 1.67, *p* = 0.200, *η*_*p*_^2^ = 0.02. Additionally, we investigated this null effect with a Bayesian repeated measures ANOVA with default prior (JASP; version 9.1: JASP Team, 2018). This allowed us to report the Bayes factor in favour of the null hypothesis that instructed switch probability did not influence classification priming at all. As influences of all other effects were already added to the null model, the reported Bayes factors can directly be interpreted as the likelihood of a null effect for the respective interaction. According to the Bayes factor (BF_01_ = 4.2) it is 4.2 times more likely that the null hypothesis is true compared to the alternative hypothesis. This is seen as substantial evidence in favour of the null hypothesis according to Jarosz and Wiley (2014).

All other effects were not significant, *F*s ≤ 3.00, *p*s ≥ 0.087, *η*_*p*_^2^ ≤ 0.04. See Tables S1 and Fig. 3 for an overview of the results.

##### Probe PE analysis

*Replication of previous effects* Similar to the RT results, we also replicated the main effects of Pfeuffer et al. (2017) in the error rates: participants committed more errors after classification switches (*M* = 9.7%) compared to classification repetitions (*M* = 5.7%), *F*(1,76) = 185.03, *p* < 0.001, *η*_*p*_^2^ = 0.71. Analogously, participants committed fewer errors after action repetitions (*M* = 7%) compared to action switches (*M* = 8.4%), *F*(1,76) = 20.56, *p* < 0.001, *η*_*p*_^2^ = 0.21. The main effect of prime type was significant, *F*(1,76) = 5.82, *p* = 0.018, *η*_*p*_^2^ = 0.07, with more errors after executed (*M* = 8.1%) compared to verbally coded primes (*M* = 7.3%).

Replicating previous findings (e.g., Pfeuffer et al., 2017), prime type interacted with classification priming, *F*(1,76) = 32.30, *p* < 0.001, *η*_*p*_^2^ = 0.30. Classification priming was more pronounced after executed primes (*M*_Priming_ = 5.6%) compared to verbally coded primes, (*M*_Priming_ = 2.5%), *t*(77) = 5.72, *p* < 0.001, *d* = 0.65. There was no significant interaction of prime type and action priming, *F*(1,76) = 1.91, *p* = 0.171, *η*_*p*_^2^ = 0.02.

Unexpectedly, action priming effects were stronger for the SC group than for the SA group, *F*(1,76) = 4.77, *p* = 0.032, *η*_*p*_^2^ = 0.06, with significant action priming in both the SC group (*M*_Priming_ = 1.93%), *t*(37) = 4.58, *p* < 0.001, *d* = 0.74, and in the SA group (*M*_Priming_ = 0.7%), *t*(39) = 1.71, *p* = 0.048, *d* = 0.27.

*Modulation by instructed switch probability* Surprisingly, there was an additional main effect of instructed switch probability, F(1,76) = 5.09, p = 0.027, *η*_*p*_^2^ = 0.06, with higher error rates after instructed 25% switch probability (M = 8%) compared to instructed 75% switch probability (M = 7.4%).  In accordance with our key hypothesis, action priming was modulated by instructed switch probability, *F*(1,76) = 5.78, *p* = 0.019, *η*_*p*_^2^ = 0.07 (see Fig. 3). As hypothesized, action priming was more pronounced under instructed 25% switch probability (*M*_Priming_ = 1.9%), *t*(77) = 5.22, *p* < 0.001, *d* = 0.59, compared to 75% switch probability (*M*_Priming_ = 0.68%), *t*(77) = 1.73, *p* = 0.043, *d* = 0.20. Surprisingly, this was further qualified by an interaction with classification priming (three-way interaction of instructed switch probability × classification priming × action priming), *F*(1,76) = 4.77, *p* = 0.032, *η*_*p*_^2^ = 0.06. In detail, classification switches reduced action priming after instructed 75% switch probability (classification repetition: *M*_Priming_ = 1.6%; classification switches: *M*_Priming_ = − 0.2%), compared to 25% switch probability (classification repetition: *M*_Priming_ = 1.5%; classification switches: *M*_Priming_ = 2.3%), *t* = 2.19*, p* = 0.017*, d* = 0.25*.*

Contrary to probe RTs, error rates showed a significant four-way interaction of instructed switch probability × classification priming × action priming × prime type, *F*(1,76) = 5.87, *p* = 0.018, *η*_*p*_^2^ = 0.07. This reflected that the previously described three-way interaction of instructed switch probability × classification priming × action priming was more pronounced for executed primes compared to verbally coded primes. However, classification switches reduced action priming effects after instructed 75% switch probability (but not after 25%) for executed primes and this pattern did not significantly differ for verbally coded primes, *t* = 1.65, *p* = 0.052, *d* = 0.19. The five-way interaction of all factors just missed significance, *F*(1,76) = 3.96, *p* = 0.050, *η*_*p*_^2^ = 0.05. All other main effects and interactions were not significant, *F*s ≤ 1.91, *p*s ≥ 0.171, *η*_*p*_^2^ ≤ 0.02.

#### Post-experimental questionnaire

As a manipulation check, we asked participants about their subjectively experienced switch probabilities following the presentation of the different switch probability cues. Specifically, we asked the participants: How often did the action/the classification really switch between the first and second time the picture was presented for the orange frame/for the blue frame? We asked participants to report the proportion of trials they estimated this to be the case.

Fourteen out of 78 participants reported at the end of the experiment that the experienced switch probability was 50% for both instructed probabilities, while the other participants reported different switch probabilities for the cues according to the instructed probabilities. We conducted an additional analysis without these participants and present the results in the section ‘Pooled analyses’.

### Discussion

Replicating the previously reported basic findings (Pfeuffer et al., 2017), we found SC priming effects after both executed and verbally coded primes. We also replicated the interaction of prime type and classification with larger SC priming effects in blocks with executed primes compared to blocks with verbally coded primes. Furthermore, we found action priming effects and an interaction of action priming and prime type. In agreement with Pfeuffer, Hosp et al. (2018), we found action priming effects in executed blocks, but no significant action priming effects in verbally coded blocks (see, however, e.g., Pfeuffer et al., 2017, for evidence of significant action priming for both prime types). Thus, our results match previous findings suggesting that action priming effects are relatively weak, especially after  verbally coded primes.

Concerning our key hypothesis, only action priming was modulated by instructed switch probability. This contrasts with a previous study (Pfeuffer et al., 2020) on experienced switch probability in which SC priming effects were found to be modulated by experienced SC switch probability. This is especially surprising, as SC priming effects are stronger than SA priming effects (e.g., Pfeuffer et al., 2017, 2020). Theoretically, it should have been easier to find an interaction of classification priming and instructed switch probability. Interestingly, there was no significant three-way interaction between group, instructed switch probability and action priming (or/and classification priming), but merely a two-way interaction of action priming and instructed switch probability. This suggests that participants processed instructed switch probability not on the level of specific SC/SA components, but rather on a more global level. That is, participants might not have clearly differentiated between switch probabilities regarding SC and SA mappings, but rather have tried to generally memorize whether stimulus mappings would repeat versus switch. In fact, it seems reasonable to assume that the overall task complexity was quite challenging for most participants. However, switch probability information was not necessary for successful task performance. Thus, it seems a plausible strategy to reduce cognitive load by processing the instructed switch probability cue as a more general indicator of switch probability rather than using it to specifically prepare for switches/repetitions in classification or action components. Alternatively, it might be that participants of both groups used instructed switch probability for the manual response of SA priming. This might have been easier, as a switch in the manual response (left vs. right) was easier to prepare for than a switch in classification (small/large vs. mechanic/non-mechanic).

Furthermore, modulations of SA priming effects could only be observed in executed blocks. The general absence of SA priming effects after verbally coded prime trials prevented us from drawing conclusions about modulations of verbally coded SA associations by instructed switch probability. One possible factor affecting the size (and thereby detectability) of SA priming effects especially after verbally coded prime trials, when SA priming effects are relatively weak, is hierarchical retrieval. In the present study, action priming effects were only present (RTs) or more pronounced (PEs) in classification repetition trials, suggesting hierarchical retrieval of classification and action components (i.e., S → C → A). In the majority of previous studies (e.g., Horner & Henson, 2011, 2012; Moutsopoulou et al., 2015; Pfeuffer et al., 2017), this interaction was reported to be non-significant. However, one previous study investigating the influence of *experienced* switch probability also reported an interaction of classification and action priming in agreement with our finding (Pfeuffer et al., 2020). In line with our results, they found that SA priming effects were only significant after classification repetitions for both prime types. This suggests that experienced/instructed probabilities of item-specific switches might be able to change the organisation of SC and SA associations. Classification switches between prime and probe trial thus seem to diminish execution-based and verbally coded SA priming effects at least under some conditions.

Considering the ideas outlined above, in Experiment 1, conditions might have been suboptimal to observe significant expectation-based modulations of SA priming effects after verbally coded prime trials as SA priming effects were generally relatively weak to begin with. The rationale of Experiment 2 was therefore to strengthen SA priming effects by exclusively realizing classification repetition trials. This modification was hypothesized to yield a modulation of SA priming effects by instructed switch probability not only following executed prime trials but also following verbally coded prime trials.

## Experiment 2

The second experiment was designed with two goals in mind: First, we wanted to confirm the results reported in Experiment 1 regarding the impact of instructed switch probability on SA priming effects. Second, we wanted to further investigate if instructed switch probability not only modulates execution-based SA priming effects (as in Experiment 1) but also verbally coded SA priming effects (i.e., the encoding/retrieval of the corresponding SA associations)—under more optimal circumstances. To do that, we changed aspects of Experiment 1 that might have weakened SA priming effects in general as outlined in the discussion above.

Therefore, to create a setting that maximizes  SA priming effects, we eliminated classification switches between prime and corresponding probe trial (i.e., SC mappings were always item specifically repeated). First, this increased the number of trials per condition. Second, as detailed before, it is possible that the retrieval of SA associations might have depended on whether SC mappings repeated. That is, if the retrieval of SA associations depended on SC associations under some conditions due to a hierarchical structure of SC and SA associations (S → C → A), it might be that under conditions that support such a hierarchical structure, SA priming effects are only observed when the classification repeats but not when it switches. Excluding item-specific switches in SC mapping therefore ensured that hierarchical structures of SC and SA associations could not impact the pattern of results observed for SA priming effects.

For Experiment 2, we also eliminated the between-subject factor. All participants were instructed that responses could change between the first and the second presentation of an image and that the cues predicted whether the probability of an item-specific switch was 25% or 75%.

### Methods

#### Participants

The sample size of the second experiment was determined based on the SA group in the first experiment (i.e., *N* = 40). Assuming a comparable effect size for the interaction of SA priming and instructed switch probability as in the first experiment (*f* = 0.369) a sample size of 40 subjects is sufficient to gain a power above 80% at an alpha level of 5% (Faul et al., 2007). This identical sample size enabled us to compare effects in Experiment 1 and 2 and attribute similarities and differences in action priming to the changes we made in Experiment 2.

We recruited 44 new participants who provided written informed consent. Due to technical problems, we lost one dataset. One participant aborted the experiment. Two participants were excluded due to insufficient performance (error rates > 3 SDs above mean), leaving a final sample of 40 participants (25 female, 15 male, 5 left handed, mean age = 25.0, SD = 4.5, range 19–37).

For a large sample size confirmation of our findings, we additionally pooled the data of the SA group in the first experiment and the data of the second experiment.

#### Stimuli and procedure

Experiment 2 was the same as Experiment 1 except that item-specific classifications always repeated (i.e., there were more trials per action condition). Note that there were, however, still two possible classification tasks (size and mechanism) that could repeat/switch on a trial-by-trial basis. Accordingly, we only tested one group, the SA group, which was instructed that responses could change with a certain probability depending on the coloured frame presented. Again, we instructed participants that item-specific mappings (classification/action) during prime trials should be remembered for recall. The experiment consisted of 80 blocks (40 executed, 40 verbally coded) and was otherwise identical to Experiment 1.

#### Data analyses

Analyses were conducted using SPSS Statistics version 27 (IBM SPSS Statistics, Armonk, NY). Performance data from both classifications (size and mechanic) and both manual responses (left and right) used in each condition was pooled. In all statistical analyses reported, an alpha level of 0.05 was used.

Prime trial analysis can be found in the supplementary material.

Probe trial RTs and PEs were submitted to a 2 × 2 × 2 repeated measures analysis of variance (ANOVA) with the within-subject factors prime type (executed vs. verbally coded), instructed switch probability (25% vs.75%) and action priming (repetition vs. switch).

### Results

#### Probe trial analysis

**Fig. 4:**
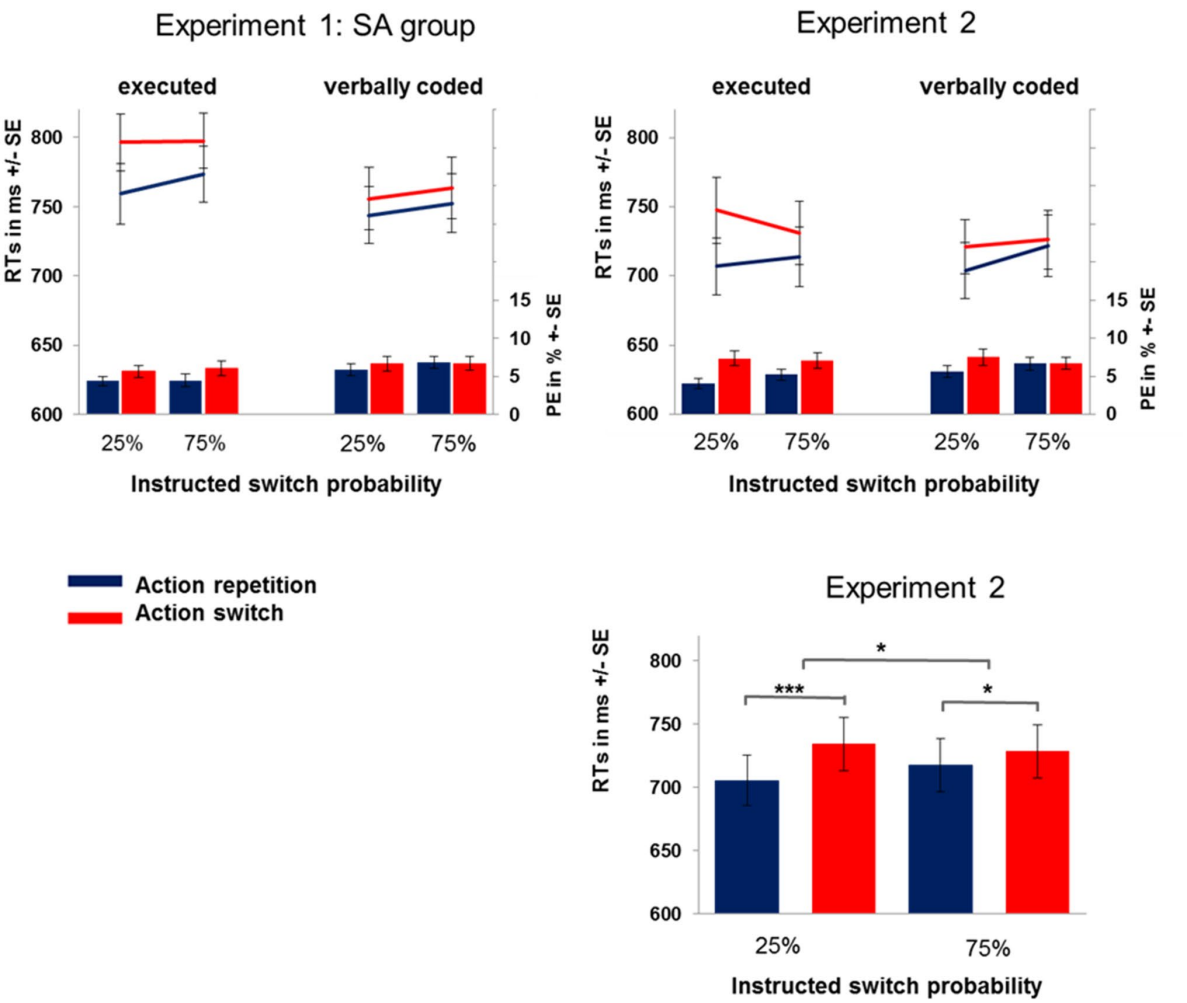
Interaction of instructed switch probability and action priming (upper panel) in Experiments 1 and 2 displayed separately for executed and verbally coded blocks. For the sake of comparison, we show only the SA group of Experiment 1 and only classification repetitions. Blue lines: action repetition reaction times (RTs), red lines: action switch RTs; blue bars: action repetition percentage of errors (PEs), red bars: action switch PEs. (Lower panel) RTs in Experiment 2 displayed together for both prime types; blue bars: action repetition RTs, red bars: action switch RTs. *** denotes *p* < 0.001 *denotes *p* < 0.05 (colour figure online)

For probe analyses, the following trials were excluded: probes of verbally coded primes with overt responses (0.3%), probes of executed primes with errors or response omissions (5.5%), and probes with response omissions (0.8%). For RT analysis, erroneous probes (6.5%) and outliers (1.0%, RTs more than 3 SDs below/above the individual cell mean) were excluded. In total, 13.0% of probe trials were excluded. An overview of the results can be seen in the Tables S1 in the Supplementary Material and in Fig. 4 upper panel.

##### Probe RT analysis

*Replication of previous effects* Replicating previous findings, we found a significant main effect of action priming, *F*(1,39) = 18.42, *p* < 0.001, *η*_*p*_^2^ = 0.32, with faster responses after action repetitions (*M* = 711.6 ms) compared to action switches (*M* = 731.5 ms). The interaction of prime type and action priming failed to reach significance, *F*(1,39) = 3.66, *p* = 0.063, *η*_*p*_^2^ = 0.086. Note, however, that a test of the directed hypothesis via an one-tailed *t*-test showed significantly stronger action priming effects after executed primes than after verbally coded primes, *t*(39) = 1.94, *p* = 0.030, *d* = 0.31, and that based on results of Experiment 1, we wanted to be sure, that action priming effects are significant after both prime types. Indeed, there was a significant priming effect after executed primes (*M*_Priming_ = 29.1 ms), *t*(39) = 4.02, *p* < 0.001, *d* = 0.64. Importantly, in contrast to Experiment 1 and as hypothesized, we found a significant priming effect also after verbally coded primes (*M*_Priming_ = 11.2 ms), *t*(39) = 1.92, *p* = 0.032, *d* = 0.30.

*Modulation by instructed switch probability* Again, in accordance with our key hypothesis, action priming interacted with instructed switch probability, *F*(1,39) = 5.20, *p* = 0.028, *η*_*p*_^2^ = 0.12 (see Fig. 4 lower panel). There were significant priming effects for instructed 25% switch probability (*M*_Priming_ = 29 ms), *t*(39) = 5.09, *p* < 0.001, *d* = 0.31, which were reduced, but still significant for 75% switch probability (*M*_Priming_ = 11 ms), *t*(39) = 1.69, *p* = 0.049, *d* = 0.026.

In contrast to Experiment 1, there was indeed, as hypothesized, no significant three-way interaction of action priming, instructed switch probability, and prime type in Experiment 2, *F*(1,39) < 1. Hence, statistically, instructed switch probability indistinguishably modulated action priming for both prime types. We additionally investigated this theoretically relevant null effect with a Bayesian repeated measures ANOVA with default prior (JASP; version 9.1, JASP Team, 2018). This allowed us to report the Bayes factor in favour of the null hypothesis that instructed switch probability did influence executed and verbally coded action priming in the same way. As influences of all other effects were already added to the null model, the reported Bayes factors can directly be interpreted as the likelihood of null effects for the respective effects. The observed Bayes factor of BF_01_ = 4.3 means that the data provides substantial evidence in favour of the null hypothesis (Jarosz & Wiley, 2014). The null hypothesis is 4.3 times more likely than the alternative hypothesis.

All other effects were not significant, *F*(1,39) ≤ 1.81, *p* ≥ 0.187, *η*_*p*_^2^ ≤ 0.04.

##### Probe PE analysis

*Replication of previous effects* There was again a main effect of action priming, *F*(1,39) = 12.21, *p* = 0.001, *η*_*p*_^2^ = 0.24, reflecting that participants committed more errors when actions item specifically switched (*M* = 7.1%) compared to when they repeated (*M* = 5.4%). Action priming did not significantly interact with prime type, *F*(1,39) = 2.73, *p* = 0.107, *η*_*p*_^2^ = 0.07, suggesting similar action priming in executed and verbally coded blocks.

*Modulation by instructed switch probability* The interaction of instructed switch probability and action priming failed to reach significance, *F*(1,39) = 3.22, *p* = 0.080, *η*_*p*_^2^ = 0.08. However, the directed (one-tailed) test according to our hypothesis predicting increased action priming under instructed 25% switch probability (*M*_Priming_ = 2.6%) versus instructed 75% switch probability (*M*_Priming_ = 0.9) was significant, *t*(39) = 1.8, *p* = 0.035, *d* = 0.28.

As for RTs, the interaction of action priming, instructed switch probability, and prime type was non-significant, *F*(1,39) < 1 and the Bayesian repeated measures ANOVA yielded substantial evidence for the null hypothesis (BF_01_ = 3.6) that instructed switch probability did modulate action priming similarly for executed and verbally coded primes (see Jarosz and Wiley, 2014).

All other effects were not significant, *F*(1,39) ≤ 1.44, *p* ≥ 0.237, *η*_*p*_^2^ ≤ 0.04.

#### Post-experimental questionnaire

To assess participants’ explicit knowledge about the actual switch probability (50%), after completion of the experiment, we asked participants about their subjectively experienced switch probabilities during probe trials for each of the two instructed switch probability cues. Four out of 40 participants reported that they experienced a switch probability of 50%. The other participants reported different switch probabilities for the cues according to the instructed probabilities.

#### Pooled analyses

For a confirmatory analysis with a larger sample, we pooled the data of the SA group in Experiment 1 (only classification repetitions) and the data of Experiment 2. Therefore, the pooled sample comprised 80 participants. Exclusion criteria were the same as before.

##### Probe RT analysis

*Replication of previous effects* As in both Experiments 1 and 2, we found a significant main effect of prime type, *F*(1,79) = 6.12, *p* = 0.016, *η*_*p*_^2^ = 0.08, with faster responses in probe trials in verbally coded blocks (*M* = 735.2 ms) compared to executed blocks (*M* = 752.2 ms). Again, action repetitions (*M* = 733.9 ms) were significantly faster compared to action switches (*M* = 753.5 ms), *F*(1,79) = 27.77, *p* < 0.001, *η*_*p*_^2^ = 0.26. In accordance with Experiment 1, prime type and action priming interacted, *F*(1,79) = 16.54, *p* < 0.001, *η*_*p*_^2^ = 0.17. Action priming was stronger in executed (*M*_Priming_ = 32.0 ms), *t*(79) = 6.20, *p* < 0.001, *d* = 0.70, compared to verbally coded blocks (*M*_Priming_ = 7.2 ms), *t*(79) = 1.59, *p* = 0.055, *d* = 0.18. This difference between prime types was significant, *t*(79) = 3.92, *p* < 0.001, *d* = 0.43.

*Modulation by instructed switch probability* Most importantly, instructed switch probability modulated action priming, *F*(1,78) = 5.52, *p* = 0.021, *η*_*p*_^2^ = 0.07. Instructed 25% switch probability (*M*_Priming_ = 26.8 ms) led to significantly stronger action priming compared to instructed 75% switch probability (*M*_Priming_ = 12.4 ms).

The three-way interaction of prime type, instructed switch probability and action priming was not significant, *F*(1,78) < 1, in accordance with Experiment 2. The Bayesian repeated measures ANOVA of the pooled data yielded substantial evidence for the null hypothesis (BF_01_ = 6.9) for this three-way interaction (see Jarosz & Wiley, 2014). The null hypothesis (prime type does not influence the interaction of action priming and instructed switch probability) was 6.9 times more likely than the alternative hypothesis.

##### Probe PE analysis

*Replication of previous effects* Similar to the RT results, we replicated the main effects of prime type, *F*(1,79) = 8.38, *p* = 0.005, *η*_*p*_^2^ = 0.10, and action priming, *F*(1,79) = 15.18, *p* < 0.001, *η*_*p*_^2^ = 0.16. Participants committed more errors in verbally coded blocks (*M* = 6.8%) compared to executed blocks (*M* = 5.6%) and in action switch (*M* = 6.9%) compared to action repetition trials (*M* = 5.5%). Again, prime type and action priming interacted, *F*(1,79) = 4.37, *p* = 0.040, *η*_*p*_^2^ = 0.05, with larger action priming effects in executed (*M*_Priming_ = 2.2%) compared to verbally coded trials (*M*_Priming_ = 0.7%).

*Modulation by instructed switch probability* As in Experiment 2, the interaction of instructed switch probability and action priming just failed to reach significance, *F*(1,79) = 3.44, *p* = 0.068, *η*_*p*_^2^ = 0.04. However, again the directed test according to our hypothesis predicting increased action priming for instructed 25% switch probability (*M*_priming_ = 2.1%) versus instructed 75% switch probability (*M*_Priming_ = 0.8%) was significant, *t*(39) = 1.85, *p* = 0.034, *d* = 0.21.

As before, the three-way interaction of prime type, instructed switch probability and action priming was not significant, *F*(1,79) < 1. The Bayesian repeated measures ANOVA comparing the null model with all two-way interactions against the alternative model with the three-way interaction yielded (according to Jarosz & Wiley, 2014) substantial evidence in favour of the null hypothesis (BF_01_ = 5.5).

To rule out that participants, who did know the discrepancy between experienced and instructed switch probability at the end, affected the results, an additional analysis, only with participants that reported experienced probabilities in accordance with the instructed switch probability (*N* = 70), was performed. This analysis yielded the same pattern of RT results. For error rates, the interaction of prime type and action priming missed significance, *F*(1,69) = 3.90, *p* = 0.052, *η*_*p*_^2^ = 0.05. As reported before, instructed switch probability did not significantly interact with action priming, *F*(1,69) = 2.58*, p* = 0.113*, η*_*p*_^2^ = 0.04. In contrast to the analysis including participants who noticed actual switch probabilities during the experiment, the post hoc test was also not significant, *t*(69) = 1.39, *p* = 0.084, *d* = 0.17.

### Discussion

First, we could confirm the results reported in Experiment 1 regarding the impact of instructed switch probability on SA priming effects. Instructed 25% switch probability led to significantly stronger SA priming effects in RTs and in PEs as compared to instructed 75% switch probability.

Second, different from Experiment 1, SA priming effects were observed not only following executed primes, but this time also following verbally coded primes. This confirms our impression that factors such as the formation of hierarchical stimulus–classification–action associations might have weakened especially verbally coded SA priming effects in Experiment 1.

Most importantly and again different from Experiment 1, instructed switch probability indistinguishably modulated action priming following executed prime trials and now also following verbally coded prime trials. The significant modulation of SA priming effects and instructed switch probability by prime type in Experiment 1 was likely caused by a general lack of SA priming effects in verbally coded blocks. In this respect, the finding in Experiment 2 and the pooled analyses that prime type did not modulate the interaction between SA priming effects and instructed switch probability is theoretically interesting, as it suggests that the executed and verbally coded SA priming effects were similarly modulated by expectations. This is in line with Pfeuffer et al. (2020) who found that experienced switch probability also indistinguishably modulated execution-based and verbally coded priming effects.

In sum, the significant modulation of SA priming effects by instructed switch probability, together with the missing modulation by prime type in Experiment 2 and in the pooled analyses, suggests similar modulation of executed and verbally coded action priming by instructed switch probability. This suggests that item-specific SA priming effects are modulated both by expectations induced based on own previous experiences (Pfeuffer et al., 2020) as well as based on mere instruction (the present experiments).

## General discussion

The key novel finding obtained in both experiments was that SA priming effects resulting from SA associations were modulated by instructed switch probability (i.e., switch expectation) in the absence of actual differences in experienced switch probabilities (Exp. 1 and Exp. 2). This modulation occurred irrespective of whether SA associations were formed under conditions of active response execution (i.e., executed blocks) or verbal coding (i.e., verbally coded blocks; see Discussion of Exp. 2).

Another important aspect of our results is that the expectation-based modulation of SA priming occurred under conditions of frequently changing instructions regarding the to-be-expected switch probability. Therefore, we show that expectations can flexibly influence SA priming effects within subject on a short timescale (as compared to Pfeuffer et al., 2020, who manipulated experienced switch probability between participants and found effects only across an entire experiment after numerous induction blocks).

Furthermore, we extend earlier results which demonstrated the modulation of SA priming effects as a function of experienced switch probabilities (Pfeuffer et al., 2020). In the present study, we show in an item-specific priming paradigm that SA priming effects can be modulated not just by experienced switch probabilities (Pfeuffer et al., 2020), but also based on expected switch probabilities induced by mere instruction. Interestingly, at the same time our findings reveal a major difference between the impact of experienced and merely instructed expected switch probabilities: while SA priming effects are similarly affected by expectations based on both experience and instruction, SC priming effects are apparently only affected by expectations  based on experience but not based on instruction alone. Based on these findings, we will next discuss the possible mechanisms underlying the modulation of SA priming effects (i.e., the encoding and/or retrieval of SA associations) by instructed as compared to experienced switch probability (i.e., explicit knowledge vs. experience).

### Instructed vs. experienced switch probability

In our study, instruction-based switch expectation and actually experienced switch probability were de-confounded by explicitly instructing the expected switch probability for the current block, while experienced switch probability in the probe trials was held constant at 50% across the entire experiment. Previous studies demonstrated that not only contrary experiences (Entel et al., 2014), but also explicit knowledge of true switch probabilities (Desender, 2018) might counteract the expected switch probability. We therefore assessed at the end of the experiment whether participants were aware of the discrepancy between experienced and expected switch probability. If they were not, a crucial influence of explicit knowledge regarding actual switch probabilities could be ruled out. Across experiments, only 18 (out of 118) participants were aware that, contrary to instructions, the actual switch probability was 50%. However, analyses without these participants revealed equivalent results.

In contrast to Pfeuffer et al. (2020) who found selective modulatory effects of experienced SC/SA switch probability (between-subject) on corresponding SC/SA priming effects, we exclusively found a modulation of SA priming (within-subject) by merely instructed SA switch probability (and SC switch probability, see Discussion of Experiment 1). In other words, it seems that both SC and SA priming effects can be modulated by expectations based on experienced probabilities (Pfeuffer et al., 2020). In contrast, only the encoding and/or retrieval of SA, but not SC components, seems to be modulated by expectations derived from instruction and explicit knowledge. Consequently, the susceptibility of SC associations to expectations derived from experience (Pfeuffer et al., 2020), but not to expectations derived from mere instruction and explicit knowledge (the present experiments), suggest key differences between the mechanisms underlying the effects of the respective expectations on the encoding and/or retrieval of SC/SA associations.

In other research fields, expectations derived from experience versus instruction were investigated, for instance, in the context of description–experience gap in risky decision making (Dutt et al., 2014; Hau et al., 2008; Hertwig & Erev, 2009; Park et al., 2021). More specifically, experience-based decision making seems to be influenced by the recency of experienced probabilities in contrast to decisions based on descriptions (i.e., instructions; e.g., Wulff et al., 2018). Future research might systematically investigate if, for instance, the recency of experienced SC switches affects item-specific SA/SC priming effects.

Lastly, we provide converging evidence that, like in the study of Pfeuffer et al. (2020), execution-based and verbally code-based SA priming was similarly modulated by expectations induced by mere instruction (i.e., by explicit knowledge). However, a possible limitation of the present study is that participants could have performed micromovements, imagined movements, or have subvocally rehearsed verbal codes during a proportion of verbally coded prime trials. Although verbally coded primes with actual key press were excluded, participants could still have unintentionally performed covert movements on a proportion of trials. Previous studies did already mention this limitation (e.g., Cohen-Kdoshay & Meiran, 2007; Liefooghe et al., 2012; Longman et al., 2019; Oshrit & Meiran, 2009). Indeed, it is not quite clear yet how verbal codes lead to the formation of SR associations.

Recent studies suggest that motor imagery may be involved or that motor imagery might at least yield similar SC/SA associations (Liefooghe et al., 2021; Palenciano, 2021; Pfeuffer et al., 2017). For this reason, future studies will have to determine the exact mechanisms leading to verbally coded SC/SA priming effects.

### Possible mechanisms for the modulation of SA priming effects

Based on previous studies, diverse mechanisms could explain how expectation based on instruction may have influenced SA associations (e.g., Bugg et al., 2015; Desender, 2018). As an example, Bugg et al. (2015) investigated the effect of expectation on cognitive control in a Stroop task. However, in four experiments the instructed expectation was consistent with the experienced proportion of congruent or incongruent trials. Therefore, experience-based and instruction-based expectation could not be differentiated. Only in the last experiment, the instructed proportion of congruent/incongruent trials conflicted with the experienced proportion of 50% congruent trials. Indeed, instructed expectation of 80% congruent trials in an upcoming list (compared to 80% incongruent or 50% congruent) of trials led to larger Stroop effects (especially when only looking at the first trial of a new block). The authors suggest that participants relaxed control mechanisms based on instructed expectation, thereby increasing facilitation and/or interference. The findings of this study seem similar to the present study, although Bugg et al. (2015) did not modulate SR associations and corresponding item-specific priming effects by expectation but rather congruency effects. Nevertheless, future experiments should investigate whether a similar explanation of relaxed control mechanism can account for the present findings in item-specific priming. At present, we cannot yet provide confirmatory evidence that a relaxation of control is indeed the mechanism underlying our finding that instruction-based expectations modulate item-specific priming effects. However, based on our results, we can exclude some potential alternative explanations regarding how our manipulation of expectations based on instructed switch probability might have modulated SA priming effects, specifically, implicit learning, adjustments of the response threshold, and intentional inhibition/reversal.

First, as elaborated on in the Introduction, the modulation of SA priming effects in our study cannot be explained by implicit learning mechanisms for two reasons: On the one hand, each item was presented only twice (once in a prime and once in a probe trial) preventing item-specific learning of switch probability in contrast to previous studies (Crump & Logan, 2010; Leboe et al., 2008). On the other hand, in contrast to Pfeuffer et al. (2020) and studies investigating the effect of instructed switch probability on response conflict (Bugg et al., 2015; Entel et al., 2014), there were no systematic differences in experienced switch probabilities in our study. That is, the actual switch proportion was always 50% for both cues irrespective of instructed switch probabilities. By de-confounding instructed and experienced switch probability in our study, we were able to exclude implicit learning mechanisms as an explanation for the influence of instructed switch probability (and resulting expectations) on SA priming effects (i.e., the encoding and/or retrieval of SA associations).

Second, it might be argued that differences in expectation based on instruction could have affected the response threshold. Indeed, Desender (2018) investigated the influence of instructed proportion congruent on congruency effects in a response conflict paradigm (Simon task) and argued that either a modulation of response threshold or strategical allocation of attention could explain the effects of instructed proportion congruent on congruency effects. He used two different instructions to disentangle possible mechanisms underlying instructed proportion congruent effects. On the one hand, Desender instructed more conservative or more liberal response thresholds focussing on accuracy or speed. On the other hand, he instructed participants that congruent/incongruent trials would be more frequent overall. Both instructions did modulate the size of congruency effects. Desender argued (based on the results of a diffusion model analysis) that the instructed proportion congruent led participants to strategically allocate more attention in incongruent trials. In the case of the present experiments, we have good reasons to rule out the speed–accuracy trade-off (i.e., a criterion shift) as an alternative explanation. If we assume that participants used block-wise instructed switch probability for responding faster/less accurate (instructed 25% switch probability) or slower/more accurate (instructed 75% switch probability), RTs and error rates should have been affected overall and result patterns in RTs and error rates should have been in opposite directions. Furthermore, in case of a simple speed–accuracy trade-off, we should not have observed a selective modulation of SA but not SC priming effects. See Supplementary Material for an additional LISAs analysis (Vandierendonck, 2018), taking speed–accuracy trade-offs into account that shows equivalent results.

A third possible explanation for the effect of instructed switch probability on SA priming effects would be that participants tried to ignore SA mappings with instructed 75% switch probability. However, participants were instructed to remember item-specific SA prime mappings for recall. As SA mapping recall was overall above chance level, we argue that voluntary ignoring of SA mappings is not a probable explanation for our results. Additionally, there is no significant effect of instructed switch probability on recall performance (see Appendix). A similar explanation would suggest that participants intentionally tried to encode the reverse reaction when 75% switch probability was instructed. There is one previous item-specific priming study that already studied such an intentional reversal of SA mapping: In Pfeuffer et al. (2019), participants were instructed to answer correctly or lie about the classification (i.e., intentionally reverse SA mappings). Thus, the presentation of a context (truth vs. lie) cue (similar to our instructed switch probability) during primes led to priming of either the true classification or the exact opposite classification. Pfeuffer et al. (2019) found that, irrespective of the priming context (truth vs. lie) participants associated the executed action with a stimulus. Thus, if participants in the present study had used a reversal strategy, we should have observed reversed SA priming effects in instructed 75% switch blocks. Therefore, based on our data, we also consider this explanation for the modulation of SA priming by instructed switch probability unlikely.

By excluding these potential explanations, we can conclude that merely expected switch probabilities induced by instruction are sufficient to modulate item-specific SA priming effects. In turn, this calls into question the assumed  automaticity of mnemonic processes underlying SA encoding/retrieval (Logan, 1990). Additionally, the expectation-based modulation of SA priming effects required rapid adaptation from block to block according to the current instructed switch probability, which we think is indicative of expectation-based flexibility. The exact mechanisms by which expected switch probably exerts control over SA associations cannot yet be determined based on the present study results, but they might be similar to those suggested by Bugg et al. (2015) and Pfeuffer et al. (2020). That is, a relaxation of conflict monitoring-like cognitive control process (e.g., Botvinick, 2007; Matthew et al., 2001) could support or hinder the encoding and/or retrieval of SA associations based on instructed (or experienced) switch probability.

Moreover, at present it is unclear whether expectations affected the encoding and/or retrieval of SA associations. The question whether encoding and/or retrieval of SA associations are affected by instructed switch probability needs to be investigated in further experiments to further elucidate the underlying mechanisms.

## Conclusion

Our study clearly demonstrates that SA priming effects can be modulated by expectations induced by mere instruction in the absence of differences in experiences. Influences of expectations on item-specific priming have only been reported based on previous own experienced switch probabilities before. Here, we show flexible and rapid within-subject modulations of SA priming effects based on mere instruction, that is, instruction-based cognitive control over SA associations. In contrast to cognitive control over SR associations based on experienced probabilities, only SA associations, but not SC associations were susceptible to expectation modulations by instructed switch probabilities. Together, our results indicate a distinction between cognitive control over SR associations based on own item-specific learning experiences on the one hand and based on mere instructions and explicit knowledge on the other hand.

### Electronic supplementary material

Below is the link to the electronic supplementary material.

Supplementary file1 (DOCX 53 kb)

## Data Availability

The raw data, the experimental E-Prime files as well as analyses scripts are available on OSF: https://osf.io/ckqhw/.

## Appendix

### Memory recall analysis

#### Experiment 1

Overall participants did recall 70.3% of SC and 64.2% of SA mappings presented during prime trials. Recall performance was above change level for classification mappings, *t*(77) = 12.74, *p* < 0.001, *d* = 1.43, and for action mapping, *t*(77) = 10.87, *p* < 0.001, *d* = 1.22.

Classification recall was analysed by a 2 × 2 × 2 × 2 mixed-design ANOVA with the between-subject factor group (SC vs. SA) and the within-subject factors prime type (executed vs. verbally coded), classification priming (repetition vs. switch) and instructed switch probability (25% vs. 75%).

As expected, for the recall of SC mappings, a main effect of classification was found, *F*(1,76) = 138.45, *p* < 0.001, *η*_*p*_^2^ = 0.65, with better recall after classification repetition (84.0%) compared to classification switch (56.0%). Furthermore, recall of executed (73.5%) and verbally coded primes (67%) differed, *F*(1,76) = 16.45, *p* < 0.001, *η*_*p*_^2^ = 0.18. Classification recall was not modulated by other factors, *F*s ≤ 1.81; *p*s ≥ 0.182, *η*_*p*_^2^ ≤ 0.02.

In turn, action recall was analysed by a 2 × 2 × 2 × 2 mixed-design ANOVA with the between-subject factor group, and the within-subject factors prime type, action priming and instructed switch probability. Recall for SA mappings was better after action repetition (75.0%) compared to action switch (53.5%), *F* = 98.31, *p* < 0.001, *η*_*p*_^2^ = 0.56. In contrast to SC recall, action mappings were better remembered after verbal coding (65.8%) compared to response execution (62.5%), *F*(1,76) = 4.41 *p* = 0.039, *η*_*p*_^2^ = 0.06. The four-way interaction of group, instructed switch probability, prime type, and action priming missed significance, *F*(1,76) = 3.49, *p* = 0.066, *η*_*p*_^2^ = 0.04. Action recall was not modulated by other factors, *F*s ≤ 2.94, *p*s ≥ 0.091, *η*_*p*_^2^ ≤ 0.04.

#### Experiment 2

Overall, participants did recall 89.2% of SC and 63.0% of SA mappings. Again, recall performance was above change level for classification mappings, *t*(39) = 17.15, *p* < 0.001, *d* = 2.75, and for action mapping, *t*(39) = 7.26, *p* < 0.001, *d* = 1.16.

As there were no classification switches in Experiment 2, recall of SC mappings was analysed by a 2 × 2 ANOVA with the within-subject factors prime type and instructed switch probability. Classification recall was not significantly modulated by these factors, *F*s ≤ 2.50, *p* ≥ 0.122, *η*_*p*_^2^ ≤ 0.06.

Action recall was analysed by a 2 × 2 × 2 ANOVA with the within-subject factors prime type, action and instructed switch probability. Action recall was only modulated by action, *F*(1,76) = 69.85 *p* < 0.001 *η*_*p*_^2^ = 0.64, with better performance after action repetitions (76%) compared to action switches (*M* = 50%). The interaction of prime type and instructed switch probability missed significance, *F*(1,76) = 3.45, *p* = 0.070, *η*_*p*_^2^ = 0.081. All other effects failed to reach significance, *F*s ≤ 1.96, *p*s ≥ 0.169, *η*_*p*_^2^ ≤ 0.05.
