# Supplementary material for: Instructing item-specific switch probability: expectations modulate stimulus–action priming
Source: Psychol Res. 2022 Jan 18;86(7):2195–214. doi: 10.1007/s00426-021-01641-z (PMC9470635; doi:10.1007/s00426-021-01641-z)
Supplement: Supplementary file 1 — Supplementary file1 (DOCX 53 kb) [file 426_2021_1641_MOESM1_ESM.docx]

**Supplementary Material**

**Table S1** with ANOVA results of Experiment 1 and 2

|  | **RTs Exp1** | **PEs Exp1** | **RTs Exp 2** | **PE Exp 2** |
| --- | --- | --- | --- | --- |
| Prime Type | F= 42.6 p<.001 η_p_^2^=.36* | F=5.8 p=.018 η_p_^2^=.07* | F<1 | F=1.44 p=.237 |
| Classification priming | F= 152 p<.001 η_p_^2^=.67 * | F=185 p<.001 η_p_^2^=.71 * | -- |  |
| Action priming | F=8.8 p=.004 η_p_^2^=.10 * | F=20.6 p<.001 η_p_^2^=.21 * | F=18.4 p<.001 η_p_^2^=.32* | F=12 p=.001 η_p_^2^=.24* |
| Instructed switch probability | F<1 | F=5.1 p=.027 η_p_^2^=0.06 * | F<1 | F<1 |
| Classification × action priming | F=4.4 p=.039 η_p_^2^=.06 * | F=1 p=.32 | -- | -- |
| Prime type × classification priming | F=50 p<.001 η_p_^2^=.40 * | F=32.3 p<.001 η_p_^2^=.30 * | -- | -- |
| Prime type × action priming | F=8.0 p=.006 η_p_^2^=.10 * | F=1.9 p=171 | F(1,38) =3.66 p=.063 | F(1,39)=2.7 p=.171 |
|  |  |  |  |  |
| Action priming x group | F=1.15 p=.286 | F=4.76 p=.032 η_p_^2^=.06 * | -- | -- |
| *Action x Instructed switch probability* | F<1 | F=5.78 p=.019 η_p_^2^=.07 * | F=5.2 p=.028 η_p_^2^=.12* | F=3.22 p=.08 η_p_^2^=.08 |
| *Prime type × action x instructed switch probability* | F=6.4 p=.014 η_p_^2^=.08 * | F<1 | F<1 | F<1 |
| *Instructed switch probability x Classification x Action* | F=2.1 p=.15 | F=4.77 p=.032 η_p_^2^=.06 * | -- |  |
| Prime Type x instructed switch probability x classification × action | F<.1 | F=5.87 p=.018 η_p_^2^=.07 * | -- |  |
| Group x prime type x instructed switch probability x classification × action | F(1,76)=1.4 p=.24 | F=3.96 p=.05 η_p_^2^=.05 | -- |  |

*denotes significant effects

## Prime trial analysis

If instructed switch probability influenced the encoding of SC and SA associations during prime trials this might be expressed by prolonged response times (RTs) for prime trials under 25% vs. 75% switch probability (cf. Ruge et al., 2018). We therefore tested if the expected switch probability affected RTs and percentage of errors (PEs) in executed primes based on a 2 x 2 mixed-design analysis of variance (ANOVA) including the within-subject factor instructed switch probability (25% vs.75%) and the between-subject factor group (SC vs. SA).

**Experiment 1**

For the analysis of executed primes, we excluded trials with response omissions (2.4%). For analysis of prime RTs we also excluded error trials (8.0%) and outliers (RTs deviating more than 3 SDs from the corresponding individual cell mean; 1.3%).

There was no significant modulation of prime trial RTs, *F*s ≤ 1.14, *p*s ≥ .288; η_p_^2^ ≤ .01, or PEs, *Fs* _≤_ 1, by any of our experimental manipulations.

**Experiment 2**

We analysed if instructed switch probability changed encoding of stimulus-action associations in executed primes by paired t-tests for RTs and PEs.

We excluded primes with response omission (1.4%) from the analysis of error rates. For the investigation of RTs, we excluded error trials (9.7%) and trials with RTs deviating more than 3 SD from the corresponding individual cell mean (1.4%). There was no significant difference (RTs and PEs) between instructed switch probabilities*, ts*<0.70, *ps*>.244, *ds*<0.11.

#### Pooled analysis

As before, RTs and PEs of executed prime trials were not significantly different for instructed 25% vs. 75% switch probability, *F*s<1.

***LISAS***

To test, if instructed switch probability changed speed-accuracy trade-off in probe trials, we investigated linear-integrated speed-accuracy score (LISAS, Vandierendonck, 2018). Additionally, as the congruency effects in RT and PE were in the same direction, an integrated speed-accuracy measure can be useful.

**LISAS =RT_correct_ + PE x S_RT_/S_PE_** per subject and condition

Where RT and PE are the means of the responses, S_RT_ refers to the standard deviation of correct RTs and S_PE_ refers to the standard deviation of PE.

We report LISAS, as they are by definition a balanced combination of speed and accuracy and can be interpreted as RT adapted for the number of incorrect responses.

**Experiment 1**

Similar to RTs, LISAS in Experiment 1 were modulated by prime type, *F(1,76)*=38.10, *p*<.001, *η_p_*^2^=.33, with faster responses after verbally coded prime (826.3 ms) compared to executed prime trials (868.5 ms). Classification priming, *F(1,76)*=238.45, *p*<.001, *η_p_*^2^=.76, and action priming, *F(1,76)*=18.31, *p*<.001, *η_p_*^2^=19, did modulate LISAs with faster responses after repeated mappings compared to switches. Prime type interacted with classification priming, *F(1,76)*=71.21, *p*<.001, *η_p_*^2^=48, and action priming, *F(1,76)*=7.28, *p*=.009, *η_p_*^2^=09, with stronger priming effects after executed compared to verbally coded primes. For classification priming, executed (M_priming_=98.5 ms), *t*(77)= 14.95, *p*<.001, *d*=1.69, as well as verbally coded primes (M_priming_=36.6 ms), *t*(77)=7.88, *p*<.001, *d*=0.89, led to significant priming effects. The difference of priming effects between prime types was significant, *t*(77)=8.35, *p*<.001, *d*=0.95. In contrast, for action priming only executed (M_priming_=25.8), *t*(77)=4.81, *p*<.001, *d*=.54, but not verbally coded primes (M_priming_=4.3 ms), *t*(77)=0.83, *p*>.2, *d*>0.09, led to significant effects. This difference was also significant, *t*(77)=2.71 *p*=.004, *d*=0.31.

As described for RTs, classification priming and action priming interacted, *F(1,76)*=4.40, *p*=.039, *η_p_*^2^=.06. Furthermore, there was a three-way interaction of prime type x instructed switch probability x action priming, *F(1,76)*=5.10, *p*=.027, *η_p_*^2^=.06. Instructed switch probability significantly modulated action priming effects in executed primes (M=24.4ms), *t*(77)=2.89, *p*>.003, *d*=.33, but not in verbally coded primes (M=-3.4), *t*(77)=.39, *p*>.69, *d*=0.04. In sum, the pattern is similar to RT interactions as could be expected, given that LISAS mainly represent RTs. These results do not suggest effects of a speed-accuracy trade-off. All other effects were not significant, Fs ≤ 2.97, *p*≥.089, *η_p_*^2^≤.04.

**Experiment 2**

LISAS were modulated by action priming, *F*(1,39)=22.90, *p*<.001, *η_p_*^2^=.37, with faster responses in action repetition (750 ms) compared to action switch (781ms). Prime type interacted with action priming, *F*(1,39)=4.60, *p*=.038, *η_p_*^2^=.11. There were significant priming effects after executed (M=42.8 ms), *t*(39)=4.80, *p*>.001, *d*=.76, and after verbally coded primes (M_Priming_=19.5), *t*(39)=2.44, *p*=.0095, *d*=.39.

Most importantly, instructed switch probability interacted with action priming, *F*(1,39)=9.08, *p*=.005, *η_p_*^2^=.19. After merely instructed 25% switch probability there was a significant priming effect in LISAs (48.1 ms), *t*(39)=5.72, *p*<.001, *d*=0.91, but not after instructed 75% switch probability (M=14.2 ms), *t*(39)=1.61, *p*=.055, *d*=0.44.

All other effects were not significant, *F*s ≤.1.9, *p*≥.17, *η_p_*^2^≤.05.
